# Glycosylated LGALS3BP is highly secreted by bladder cancer cells and represents a novel urinary disease biomarker

**DOI:** 10.1002/1878-0261.70140

**Published:** 2025-10-05

**Authors:** Asia Pece, Giulio Lovato, Ilaria Cela, Arianna Mercatelli, Benedetta Ferro, Jussi Nikkola, Sara Pagotto, Tommaso Grottola, Vincenzo De Laurenzi, Rossella Cicchetti, Antonio Marchetti, Luigi Schips, Rossano Lattanzio, Stefano Iacobelli, Emily Capone, Peter Black, Mads Daugaard, Michele Marchioni, Gianluca Sala

**Affiliations:** ^1^ Department of Innovative Technologies in Medicine & Dentistry “G. d'Annunzio” University of Chieti‐Pescara 66100 Italy; ^2^ Center for Advanced Studies and Technology (CAST) “G. d'Annunzio” University of Chieti‐Pescara 66100 Italy; ^3^ Diagnostic Molecular Pathology Unit of Anatomic Pathology, SS Annunziata Hospital Chieti 66100 Italy; ^4^ Department of Urologic Sciences University of British Columbia Vancouver, BC V6H 3Z6 Canada; ^5^ Vancouver Prostate Centre Vancouver V6H 3Z6 BC Canada; ^6^ Department of Medical Oral Science and Biotechnology G. d'Annunzio University Chieti 66100 Italy; ^7^ Unit of Surgical Oncology, Casa di Cura Pierangeli Pescara 65100 Italy; ^8^ Urology Unit, Department of Medical, Oral and Biotechnological Sciences ‘G. d'Annunzio University’ Chieti 66100 Italy; ^9^ MediaPharma s.r.l., Via Colonnetta 50/A Chieti 66100 Italy; ^10^ Department of Science ‘G. d'Annunzio University’ Chieti Italy

**Keywords:** bladder cancer, glycosylation, LGALS3BP, non‐muscle‐invasive bladder cancer, urinary biomarker

## Abstract

Bladder cancer incidence has recently risen, making it the ninth most diagnosed cancer, highlighting an urgent need for novel and effective diagnostic and therapeutic strategies to improve patient outcomes. Here, we report on a secreted glycoprotein, Galectin‐3‐binding protein (LGALS3BP), as a potential biomarker and therapeutic target for bladder cancer. We found a significantly elevated LGALS3BP expression in bladder cancer tissues, correlating with disease progression. Moreover, urinary and serum levels of LGALS3BP were significantly higher in patients compared to healthy individuals, with a strong correlation observed between elevated urinary protein levels and tumor grade. Of note, LGALS3BP produced by tumor cells treated with a mannosidase I inhibitor, Kifunensine, exhibited increased reactivity to a therapeutic antibody (denoted as “1959”), suggesting that glycosylation of LGALS3BP may influence antibody recognition and protein function. Furthermore, administration of 1959‐sss/DM4 antibody–drug conjugate in two xenograft mouse models of human bladder cancer resulted in complete inhibition of tumor growth. In summary, findings presented here highlight LGALS3BP as a promising candidate for further investigation into its potential as a urinary biomarker and a therapeutic target for bladder cancer.

## Graphical abstract

Urinary LGALS3BP is elevated in bladder cancer patients compared to healthy controls as detected by the 1959 antibody–based ELISA. The antibody shows enhanced reactivity to the high‐mannose glycosylated variant secreted by cancer cells treated with kifunensine (KIF). As an antibody–drug conjugate (ADC), 1959 mAb shows therapeutic efficacy in preclinical models, highlighting LGALS3BP both as a potential biomarker and therapeutic target.

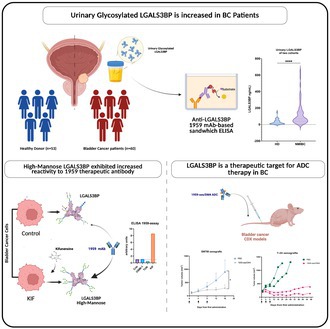

**Abbreviations**
ADC: antibody‐drug conjugate
BC: Bladder cancer
DAB: 3,3′‐diaminobenzidine
DAR: drug‐to‐antibody ratio
DMEM: Dulbecco's modified Eagle medium
DMSO: dimethyl sulfoxide
ELISA: enzyme‐linked immunosorbent assay
EV: Enfortumab vedotin
FBS: fetal bovine serum
HD: healthy donors
HG: high‐grade
H&N: head and neck cancer
HRP: horseradish peroxidase
ICD: immunogenic cell death
ICI: immune checkpoint inhibitor
IHC: immunohistochemistry
IHS: immunohistochemical score
KIF: Kifunensine
LG: low‐grade
LGALS3BP: Galectin‐3‐binding protein
MEM: Minimum essential medium
MFI: mean fluorescence intensity
MIBC: muscle‐invasive bladder cancer
MIRV: mirvetuximab soravtansine‐gynx
MTT: 3‐(4,5‐dimethyldiazol‐2‐yl)‐2,5‐diphenyl tetrazolium bromide
NDP: NanoZoomer Digital Pathology
NMIBC: non‐muscle‐invasive bladder cancer
OD: Optical density
PEA: proximity extension analysis
RPMI: Roswell Park Memorial Institute
SD: standard deviation
SEM: standard error of the mean
TUN: Tunicamycin
TURBT: transurethral resection of the bladder tumor

## 1 Introduction

Bladder cancer (BCa) is a significant global health concern, as it is one of the most common malignancies of the urinary system, with an estimated 80 000 new cases and 17 980 deaths worldwide in 2020 [1]. Its incidence has steadily risen in recent years, making it the ninth most commonly diagnosed cancer, with a corresponding increase in mortality rates [1]. BCa is classified into non‐muscle‐invasive (NMIBC) and muscle‐invasive (MIBC) based on tumor invasion depth. NMIBC accounts for approximately 70% of initial diagnoses and is typically treated with transurethral resection of the bladder tumor (TURBT). However, it carries a high risk of recurrence and progression, with about 30% of cases advancing to MIBC. Due to these risks, NMIBC patients require long‐term monitoring based on their risk group. In contrast, MIBC is associated with a greater incidence of metastasis and is treated with radical cystectomy, often combined with neoadjuvant chemotherapy to prevent tumor spread [2]. Bladder cancer can be further classified as high‐grade (HG), which is fast‐growing, invasive, and resistant to therapy; and low‐grade (LG), which is characterized by frequent recurrence but has a low likelihood of progressing to HG disease or becoming invasive [3].

Early detection of BCa is crucial for improving patient outcomes. Therefore, there is an urgent need for highly sensitive and specific noninvasive approaches to enhance cancer detection as well as cancer recurrence and spreading [4].

In this study, we explored the potential of LGALS3BP (Galectin‐3‐binding protein, also known as Mac‐2 BP or 90 K) as a novel circulating biomarker for liquid biopsy in BCa. LGALS3BP is a secreted, highly glycosylated protein implicated in various biological processes, including immune response modulation, angiogenesis, cell adhesion, migration, and tumor microenvironment interactions, making it closely associated with tumor growth and progression [5].

While LGALS3BP expression remains low in normal tissues, it is significantly elevated in various cancers as a circulating protein in body fluids such as blood and saliva, making this protein a valuable candidate for noninvasive diagnostic approaches [6, 7, 8]. We developed a custom in‐house ELISA to detect LGALS3BP in the serum and urine. This assay is based on LGALS3BP targeting 1959 antibody, which serves as the backbone of the antibody–drug conjugate (ADC) 1959‐sss/DM4, formed by conjugation to the maytansine derivative DM4. Our results show that LGALS3BP expression is significantly elevated in BCa patients compared to healthy controls. Moreover, 1959sss/DM4 ADC has demonstrated potent and sustained antitumor activity in preclinical models of bladder cancer.

This study is the first report of the contemporary measurement of LGALS3BP in urine and serum. It highlights the potential of the protein as a promising biomarker for liquid biopsy and a therapeutic target for ADC‐based treatment in BCa.

## 2 Materials and methods

### 2.1 Cell lines

A panel of eight bladder cancer cell lines including HT‐1376 (RRID:CVCL_1292), UM‐UC‐3 (RRID:CVCL_1783), HT‐1197 (RRID:CVCL_1291), SW780 (RRID:CVCL_1728), T‐24 (RRID:CVCL_0554), 5637 (RRID:CVCL_0126), TCCSUP (RRID:CVCL_1738), RT‐4 (RRID:CVCL_0036), neuroblastoma cell line SKNAS (RRID:CVCL_1700), and head and neck cancer cell line HOC621 (RRID:CVCL_8699) were purchased from American Type Culture Collection (Rockville, MD, USA). All the tumor cells were grown in complete cell culture media purchased from Gibco (Life Technologies, Toulouse, France) supplemented with 10% heat‐inactivated FBS, 100 U·mL^−1^ penicillin, and 100 μg·mL^−1^ streptomycin and incubated at 37 °C in humidified air with 5% CO_2_. HT‐1376, UM‐UC‐3, HT‐1197, TCCSUP were grown in complete MEM supplemented with 1% essential amino acids; T‐24 and RT‐4 in complete McCoy's 5A; SW780, SKNAS, and HOC621 in complete DMEM; 5637 in complete RPMI 1640. Cell lines were authenticated by the supplier (ATCC) and used for experiments within 3 months of resuscitation. All cultures were routinely monitored and confirmed to be free of mycoplasma contamination.

### 2.2 Immunohistochemistry

Formalin‐fixed, paraffin‐embedded tissue sections were immunostained using a goat polyclonal antibody against human LGALS3BP (1 : 400 dilution; 60‐min incubation; catalog no. AF2226; R&D Systems, Minneapolis, MN, USA). Antigen retrieval was carried out by microwave treatment at 750 W for 10 min in 10 mmol·L^−1^ sodium citrate buffer (pH 6.0). Signal amplification was performed using the secondary biotinylated anti‐goat LSAB kit, and 3,3′‐diaminobenzidine (DAB) was used as a chromogen. Immunostained sections were digitized using the NanoZoomer Digital Pathology (NDP) System (Hamamatsu, Welwyn Garden City, UK). An immunohistochemical score (IHS) was calculated by multiplying the percentage of tumor cells positive for LGALS3BP (0–100%) by the staining intensity, assessed on a four‐level scale: 0 (absent), 1 (mild), 2 (moderate), 3 (strong). The minimum score is “0” and the maximum is “300”. Based on the distribution of LGALS3BP immunohistochemical expression, we selected a cutoff value of 200 IHS. This value was chosen arbitrarily and corresponds to the median (50th percentile) expression of LGALS3BP in our cohort of tumor cases, allowing us to dichotomize LGALS3BP expression. This dichotomization enabled us to categorize the cases as “High” and “Low” in terms of LGALS3BP expression.

### 2.3 Western blotting analysis

To evaluate LGALS3BP protein levels, bladder cancer cells were seeded at a density of 5 × 10^5^ cells in complete medium, harvested and lysed after 48 h with RIPA buffer containing protease inhibitors cocktail and Na_3_VO_4_ (Sigma Aldrich Corporation, St. Louis, MO, USA) as phosphatase inhibitor. Lysates were clarified by centrifugation at 13 000 rpm for 10 min at 4 °C. Lysates and cell culture supernatants were subjected to 10% SDS/PAGE electrophoresis and electrotransferred to nitrocellulose membrane (Merck Millipore, Darmstadt, Germany) for western blotting analysis. The membranes were blocked with 5% nonfat dry milk in PBS with 0.1% Tween20 for 1 h at room temperature. Membranes were incubated overnight at 4 °C with anti‐human LGALS3BP (goat polyclonal; 1 : 1000, #AF2226; R&D Systems, USA) and anti‐human GAPDH (rabbit monoclonal, D16H11 clone; 1 : 1000, #5174, Cell signaling technology, Danvers, MA, USA). After three washes in PBS‐0.1% Tween20, membranes were incubated with horseradish peroxidase (HRP)‐conjugated secondary antibodies (anti‐rabbit; Biorad, Berkeley, CA, USA; anti‐goat; Invitrogen, Life Technologies, Waltham, MA, USA) at room temperature for 1 h. Signal detection was performed using Clarity Western ECL substrate (1705061; Biorad). Images of membranes were acquired with a UvitecFire reader (Cambridge, UK) and analyzed with Alliance Uvitec software (Cambridge, UK).

### 2.4 Treatment with glycosylation inhibitors

As previously described [9], T24, HOC621 (head and neck cancer), and SKNAS (neuroblastoma) cells were seeded at a density of 1 × 10^6^ in 60‐mm dishes under standard growth conditions for 24 h. Cells were then treated with 5 μm of Kifunensine (KIF) (#K1140; Sigma‐Aldrich Corporation), 10 μg·mL^−1^ of Tunicamycin (TUN) (#T7765; Sigma‐Aldrich Corporation), or 50 μm of OSMI‐1 (#SML1621; Sigma‐Aldrich Corporation) in serum‐free conditions. After 48 h, cell culture supernatants were collected, centrifuged at 3000 ***g*** for 10 min at 4 °C, and stored at −80 °C until further usage. Cells were harvested to obtain protein lysates analyzed by western blotting using anti‐human LGALS3BP (goat polyclonal; 1 : 1000, #AF2226; R&D Systems, USA) and anti‐human β‐actin antibody (mouse monoclonal, 1 : 40000, #A5441; Sigma‐Aldrich Corporation).

### 2.5 Immunofluorescence and confocal microscopy

T24 cells were plated at 30% confluence on glass coverslips and grown under standard growth conditions. After 24 h, cells were incubated with KIF (5 μm) or PBS as a control for 48 h in serum‐free conditions. Then, cells were washed with PBS and fixed with 4% paraformaldehyde. After washing with PBS, cells were incubated with PBS and 5% goat serum as a blocking solution for 30 min at RT. Cells were then stained with 10 μg·mL^−1^ of anti‐LGALS3BP 1959 antibody at 4 °C for 90 min in 5% goat serum. Finally, cells were stained with anti‐human AlexaFluor‐488 conjugated secondary antibody (1 : 100, #A11013; Invitrogen, Life Technologies) at 4 °C for 1 h and mounted using ProLong Gold Antifade Mounting with DAPI (P36935; ThermoFisher Inc., Waltham, MA, USA). Confocal images were acquired as z‐stacks using a Zeiss LSM800 inverted confocal microscope system (Carl Zeiss, Gottingen, Germany); detector gain voltages and pinhole were set at the beginning of the experiment and maintained constant during the acquisition of all samples. The z‐stacks were then processed and merged using ImageJ software. A maximum intensity projection of the green signal channel was generated, and the resulting images were used for quantification of fluorescence intensity within the software.

### 2.6 Cytotoxicity assays

Cell proliferation was assessed by MTT [3‐(4,5‐dimethyldiazol‐2‐yl)‐2,5‐diphenyl tetrazolium bromide] assay (Sigma‐Aldrich Corporation). T24 and SW780 cells were seeded in 24‐well plates at a density of 1 × 10^4^ cells per well, whereas MSC and hGF cells were seeded at a density of 1.5 and 2 × 10^4^ cells per well, respectively; all cells were then cultured under standard growth conditions for 24 h. Cells were then treated with SH‐DM4 (DM4) (Sellek Chemicals LLC, Houston, TX, USA) or Cisplatin at indicated concentrations for 72 h. At the end of the treatment, cells were incubated with 250 μL of MTT solution (serum‐free medium with 0.5 mg/mL of MTT) for a further 2 h. After the removal of MTT solution, 200 μL of dimethyl sulfoxide (DMSO) was added to the wells for 10 min, and the absorbance at 570 nm was measured using a multi‐plate reader (Tecan, Mannedorf, Switzerland). IC50 (Inhibition of cellular proliferation by 50%) values were calculated by using graphpad prism 9.0 software (GraphPad Software, Inc., CA, USA). All experiments were performed in triplicate.

### 2.7 *In vivo* studies

Athymic CD‐1 nu/nu female mice were purchased from Envigo RMS (Udine, Italy) at the age of 5–7 weeks. Mice were kept under specific pathogen‐free conditions. The room temperature was at 22 ± 2 °C and relative humidity 50 ± 15%. Cages, bedding, and food were autoclaved before use. Mice were provided with a standard diet and water *ad libitum* and acclimatized for 2 weeks before the start of the experiments. Housing and all procedures involving the mice were performed according to the protocol approved by the Institutional Animal Care and Use Committee of the Italian Ministry of Health (Authorization no. 1118/2020).

5 × 10^6^ exponentially growing T24 or SW780 cells were subcutaneously inoculated into the right flank of the mice in a total volume of 200 μL of PBS and Matrigel (ratio 1 : 3). For therapeutic studies, when tumors became palpable (approximately 100 mm ^3^ of volume), animals were randomized into 2 groups to provide a similar range of tumor sizes for each group. The treated group received intravenous injections of 1959‐sss/DM4 twice weekly for a total of three injections (10 mg·kg^−1^), whereas the control group received PBS only. The mice's weight and the tumor volume were monitored weekly by a caliper and calculated using the following formula: tumor volume (mm^3^) = (length × width^2^)/2. A tumor volume of 1 cm^3^ was chosen as a humane endpoint for all experiments, after which the mice were sacrificed. Blood was collected by intracardiac puncture immediately after the mice's sacrifice in a tube containing a glass Pasteur pipette to induce blood clotting. Serum was obtained after centrifugation of clotted blood samples at 3000 ***g*** for 15 min at 4 °C and stored at −80 °C. Tumors were collected in formalin and embedded in paraffin.

### 2.8 Enzyme‐linked immunosorbent assay (ELISA)

To quantify LGALS3BP in cell‐derived supernatants, serum, and urine samples, a custom sandwich ELISA was developed. NUNC Maxisorp 96‐well plates (Thermo Fisher Scientific) were coated overnight at 4 °C with 2 μg·mL^−1^ of humanized anti‐LGALS3BP antibody 1959 in PBS. Following a blocking step with PBS 1% (w/v) BSA for 1 h at room temperature (RT), 100 μL of either recombinant LGALS3BP standards (1–250 ng·mL^−1^), cell supernatants, diluted urine samples (1 : 10 in PBS), or controls (PBS and LGALS3BP‐depleted urine as blanks) were added to the wells and incubated for 1 h at RT. Plates were washed three times with PBS containing 0.05% (v/v) Tween‐20, followed by the addition of biotinylated anti‐LGALS3BP antibody 1959 (1.8 μg·mL^−1^) for 1 h at RT. For detection, after three washes with PBS‐0.05% Tween‐20, Streptavidin‐HRP was added (Invitrogen, #SA10001, 1 : 10000) and incubated for 1 h at RT. After washing, stabilized chromogens were added for 6 min in the dark, and the reaction was stopped with 1 N H_2_SO_4_. Absorbance was measured at 450 nm using a Tecan microplate reader (Männedorf, Switzerland).

In the supernatants of glycosylation inhibitors treated cells, LGALS3BP was measured using the Proteintech™ Human LGALS3BP ELISA Kit (Cod. KE00155) following the manufacturer's protocol.

### 2.9 Recombinant human LGALS3BP production

Recombinant human LGALS3BP was purified from serum‐free conditioned medium of human embryonic kidney EBNA‐293 cells (Invitrogen) transfected with LGALS3BP cDNA, as described previously [10]. Purification was performed using an AKTA Start FPLC system (Cytiva, Marlborough, MA, USA) and a HiTrap NHS‐Activated HP affinity column (Cytiva) pre‐conjugated with 10 mg of anti‐LGALS3BP antibody 1959. The conditioned medium was recirculated overnight at 0.5 mL·min^−1^ at 4 °C. After washing the column with 25 column volumes (CV) of 50 mm Tris–75 mM NaCl, LGALS3BP was eluted with 2 CV of IgG Elution Buffer (Thermo Fisher Scientific) at 1 mL·min^−1^. Elution fractions were neutralized with 1 M Tris/HCl (pH 8.5), pooled, concentrated using 30 kDa MWCO centrifugal filters (Merck, Darmstadt, Germany), and dialyzed into PBS. The purified protein was aliquoted and stored at −80 °C.

### 2.10 Subjects' enrollment and urine samples processing

All procedures involving human participants were conducted in accordance with the ethical standards of the institutional research committee (Comitato di Etica per la Ricerca Biomedica della Provincia di Pescara e Chieti e dell'Università degli Studi “G. D'Annunzio” di Chieti‐Pescara; protocol no. 1767), and the GU Biobank human ethics protocol from the University of British Columbia (H21‐03722). All participants were male or female adults who provided informed consent prior to enrollment.

Twenty‐six and 38 (total = 64) hospitalized patients diagnosed with bladder cancer were enrolled, respectively, at the “SS. Annunziata” Hospital (Chieti, Italy) and at the Vancouver Prostate Center (VPC, Vancouver, Canada). Urine samples were collected on the day of inclusion, prior to transurethral resection of bladder tumor (TURBT) surgery. Additionally, urine samples were collected from healthy volunteers (*n* = 53), resulting in a total of 117 samples included in this retrospective study. Detailed patients' clinical characteristics and healthy subjects' information are provided in the Tables S2–S4. Urine samples were centrifuged at 1500 ***g*** for 20 min at 4 °C. Urine supernatants were aliquoted and stored at −80 °C until analysis.

### 2.11 Immunoprecipitation of LGALS3BP from urine samples

To isolate LGALS3BP from human urine, samples (300 μL each) were first diluted 1 : 10 in phosphate‐buffered saline (PBS, pH 7.4) to minimize matrix interferences such as high salt and urea concentrations. Pre‐clearing was performed by incubating the diluted samples with 50 μg of Protein A‐conjugated agarose beads (Thermo Scientific) for 30 min at 4 °C on a rotator. Samples were then centrifuged at 12000 rpm for 30 s at 4 °C, and the supernatants (pre‐cleared urine) were collected. Immunoprecipitation was carried out by incubating ~5 μg of anti‐LGALS3BP monoclonal antibody 1959 with the pre‐cleared urine overnight at 4 °C under continuous rotation. The immune complexes were then captured by adding 100 μg of Protein A agarose beads and incubating for an additional 3 h at 4 °C. Following centrifugation at 12 000 rpm for 2 min at 4 °C, the supernatants were removed and stored as LGALS3BP‐depleted urine. The bead‐bound immunocomplexes were washed once with 1 mL of ice‐cold PBS, centrifuged again, and the pellets resuspended in 100 μL of 5× reducing Laemmli sample buffer to dissociate LGALS3BP from the antibody and incubated at RT for 5–10 min with gentle rotation. After another centrifugation, supernatants were collected and then denatured at 90 °C for 5 min. Eluted proteins were subsequently analyzed by SDS/PAGE and western blotting.

### 2.12 Statistical analysis

For ELISA data, *P* values were determined by Mann–Whitney and Kruskal–Wallis tests; for *in vivo* xenograft growth curves, *P* values were determined by the Mantel–Cox test and considered significant for *P < 0.05, **P < 0.005, and ***P < 0.0005. Experimental sample numbers (*n*) are indicated in the figure legends. Youden's J index derived from the receiver operating characteristic (ROC) curve was used to determine the optimal cutoff value for LGALS3BP expression in pathological samples, enabling discrimination between patients and healthy controls.

## 3 Results

### 3.1 LGALS3BP is highly expressed in bladder cancer tissue

Our previous studies have identified LGALS3BP as a secreted, highly glycosylated protein that is abundantly expressed in various tumors, including melanoma, head and neck (H&N) cancer, neuroblastoma, and glioblastoma [9, 11, 12, 13]. In this study, we aimed to assess the expression of the protein in bladder carcinoma. To assess LGALS3BP expression in bladder cancer tissues, we retrospectively analyzed 29 paraffin‐embedded tissue samples collected at the time of initial diagnosis from patients with urothelial carcinoma who underwent transurethral resection of bladder tumor (TURBT). Among these samples, 11 resulted in MIBC while 18 scored as NMIBC. Among MIBC, 5 of them were diagnosed at the first biopsy while 6 progressed to advanced stage in a 3‐year period.

**Fig. 1:**
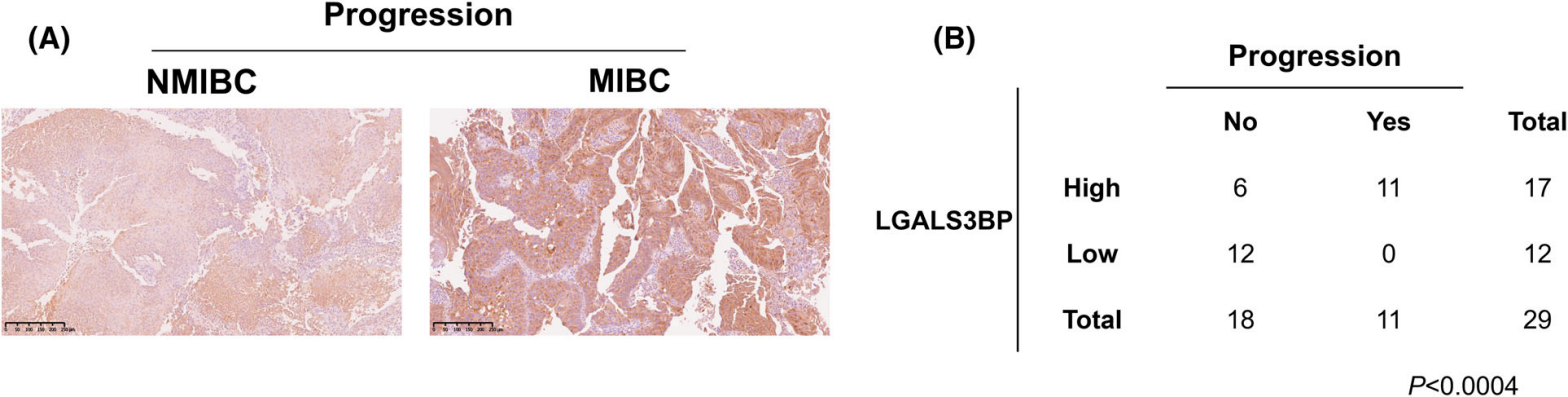
LGALS3BP is highly expressed in bladder cancer tissues. (A) Immunohistochemical analysis of LGALS3BP expression in bladder cancer tissues at first transurethral resection of the bladder tumor (TURBT). Representative images of non‐muscle‐invasive bladder cancer (NMIBC) and muscle‐invasive bladder cancer (MIBC) tissue samples are shown. Scale bar: 250 μm. NMIBC *n* = 18; MIBC *n* = 11. (B) Contingence table reporting analysis of LGALS3BP expression in patients who progressed (MIBC, *n* = 11) or not (NMIBC, *n* = 18). Patients were stratified based on expression of LGALS3BP (high or low) and progression (NMIBC *vs*. MIBC).

Overall, LGALS3BP was highly expressed across all samples (Fig. 1). However, when patients were stratified by disease aggressiveness and categorized into high and low LGALS3BP expression groups using the median value as a cutoff, we observed that 100% of MIBC patients exhibited high LGALS3BP expression at initial TURBT, compared to only 33% of NMIBC patients (Fig. 1; *P* < 0.0004). These findings indicate that LGALS3BP is not only highly expressed in bladder cancer but also correlates with more advanced disease.

While LGALS3BP expression is low in most normal tissues [13], urothelial cells have been found to be positive for LGALS3BP staining, as confirmed by the Human Protein Atlas repository (https://www.proteinatlas.org/search/LGALS3BP).

### 3.2 LGALS3BP is highly expressed in bladder cancer cell lines and represents a therapeutic target for ADC therapy

**Fig. 2:**
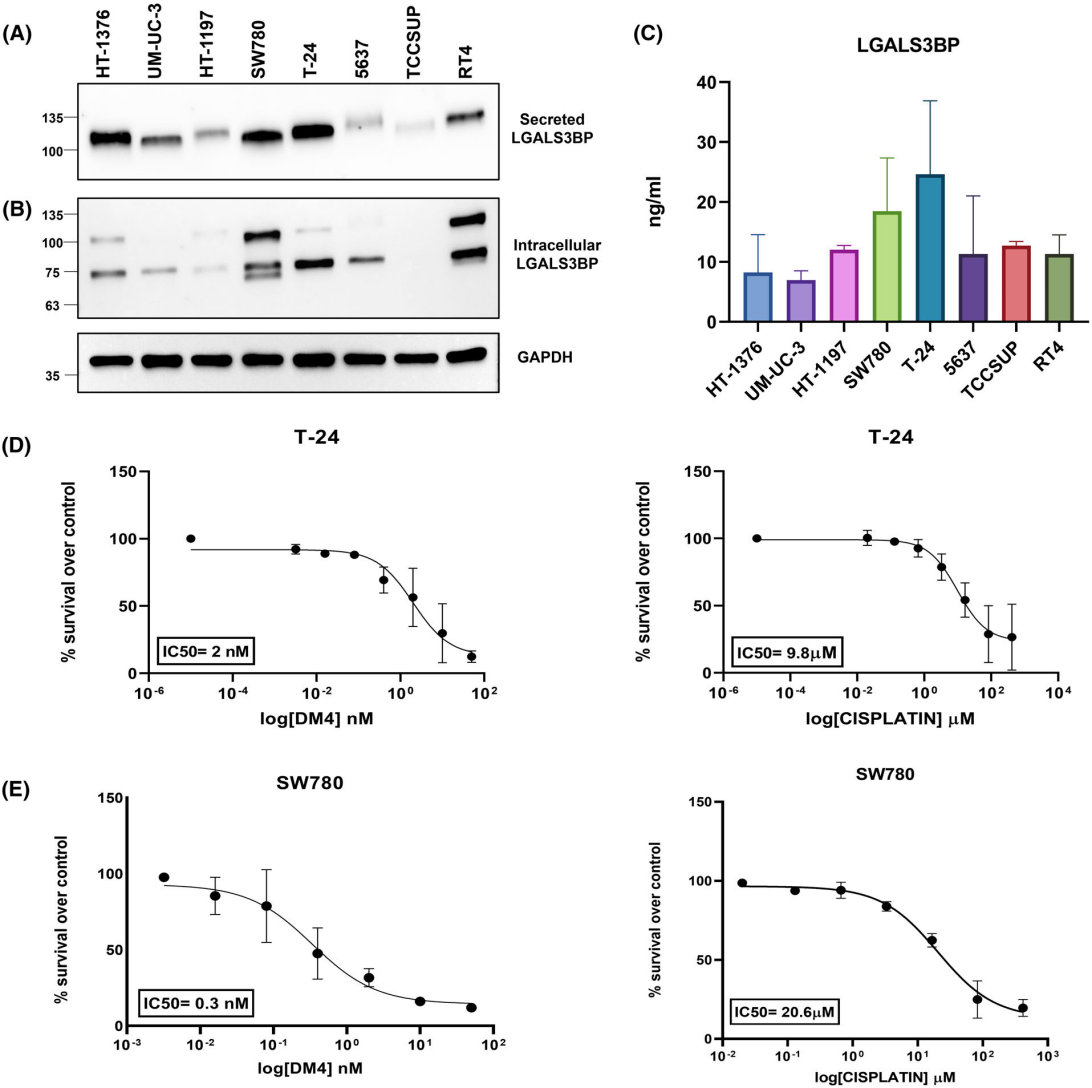
LGALS3BP expression in bladder cancer cell lines. (A) Secreted and (B) intracellular LGALS3BP protein levels in a panel of human bladder cancer cell lines analyzed through western blotting in cell culture supernatants (*n* = 2) and cell lysates (*n* = 2), respectively. Equal amounts of samples were loaded per lane. GAPDH was used as a loading control. (C) Histogram showing secreted LGALS3BP levels (ng·mL^−1^) evaluated by a 1959‐based sandwich enzyme‐linked immunosorbent assay (ELISA) (*n* = 3). Data are presented as mean ± standard deviation (SD). *In vitro* IC_50_ curves evaluated by 3‐(4,5‐dimethyldiazol‐2‐yl)‐2,5‐diphenyl tetrazolium bromide (MTT) assays by exposing (D) T‐24 (*n* = 2) and (E) SW780 (*n* = 3) cells for 72 h to increasing doses of Cisplatin and DM4. IC_50_ values are reported for each cell line considered upon different treatments. Data are presented as mean ± SD.

To better define LGALS3BP's role in bladder cancer, we moved on to cellular models. To this aim, we evaluated LGALS3BP both intracellularly and as a secreted protein in a panel of human bladder cancer cell lines (Table S1). Western blotting analysis indicated that LGALS3BP is expressed in all the cell lines tested, although the levels and the molecular size pattern varied in a cell line‐dependent manner. As shown in Fig. 2B, we detected the non‐glycosylated form of LGALS3BP protein (around 70 kDa) and the mature highly glycosylated form (around 100 kDa) in the intracellular compartment. Interestingly, only the high molecular weight form of the protein was detected in the cell culture supernatant, with slight shifts in band size, suggesting that the glycosylation pattern varies depending on the cellular model used (Fig. 2A). To quantify the expression level of the secreted protein, we developed an in‐house, sandwich‐type ELISA, utilizing the therapeutic 1959 anti‐LGALS3BP antibody (Fig. 2C). Among the different cell lines (type and origin reported in Table S1), high‐grade MIBC T‐24 and low‐grade NMIBC SW780 exhibited the highest levels of both intracellular and secreted LGALS3BP (Fig. 2A–C). Therefore, we chose these two cellular models for further studies.

### 3.3 Therapeutic activity of 1959‐sss/DM4 ADC

Next, we evaluated the potential of LGALS3BP as a therapeutic target for ADC using the 1959‐sss/DM4 ADC developed by our group. This ADC is designed using site‐specific conjugation of DM4‐SH to a modified variant of the 1959 antibody, in which the three cysteine residues of the heavy chain have been replaced with serines. This modification enables the generation of an ADC with a fixed drug‐to‐antibody ratio (DAR) of approximately 2.

For therapeutic studies, MIBC T‐24 and NMIBC SW780 xenograft models were employed. These two cell lines, that showed high LGALS3BP levels (Fig. 2A–C), exhibited a very marked sensitivity to ADC payload DM4 (IC_50_ in the nanomolar range) compared to standard chemotherapeutic agents, such as cisplatin (IC_50_ in the micromolar range) (Fig. 2D,E).

**Fig. 3:**
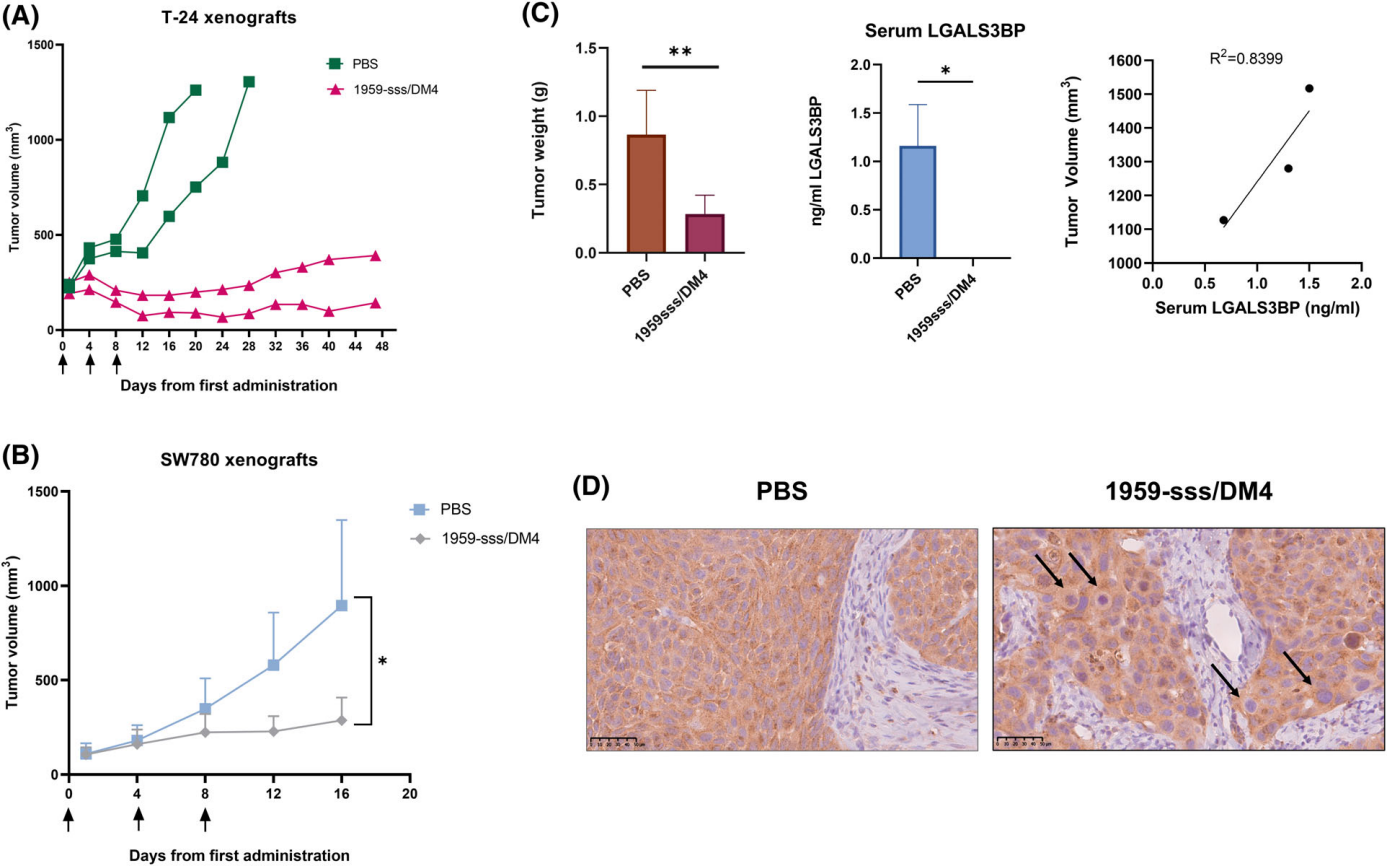
1959‐sss/DM4 antitumor activity in subcutaneous xenograft models of bladder cancer cells. (A) Single animal tumor growth expressed as tumor volume (mm ^3^) of T‐24 subcutaneous xenografts in nude mice treated with vehicle (PBS) (*n* = 2) or 1959‐sss/DM4 (10 mg·kg^−1^; twice weekly; three total i.v. injections) (*n* = 2). Arrows indicate treatment administration. Data are presented as mean ± standard error of the mean (SEM). (B) Tumor growth expressed as mean tumor volume (mm ^3^) from the first treatment of SW780 subcutaneous xenografts in nude mice treated with vehicle (*n* = 6) or 1959‐sss/DM4 (*n* = 6). Arrows indicate treatment administration (10 mg·kg^−1^; twice weekly; three total i.v. injections). Data are presented as mean ± SEM. (C) Tumor weight (g) at the endpoint of the SW780‐xenograft *in vivo* therapeutic experiment (left panel). Statistical significance was determined by *t*‐test (**P < 0.01). Commercial anti‐LGALS3BP ELISA kit showing circulating LGALS3BP levels (ng·mL^−1^) in serum derived from SW780‐xenografted mice treated with PBS or 1959‐sss/DM4 (middle panel). Correlation between circulating LGALS3BP levels (ng·mL^−1^) in PBS‐treated SW780‐xenografted mice serum and corresponding tumor volumes (mm ^3^) (right panel). Correlation value is reported (*R* ^2^ = 0.8399). (D) Representative immunohistochemistry (IHC) sections of SW780 tumors in mice treated with PBS (*n* = 6) or 1959‐sss/DM4 (*n* = 6) stained for LGALS3BP. Arrows indicate giant cells, which are the hallmarks of mitotic catastrophe. *P < 0.05. Magnification 40X. Scale bar: 50 μm.

Due to low growing efficiency we experienced with MIBC T‐24 cells, only 4 xenografts were obtained. They were randomly assigned to receive vehicle (PBS) or ADC 1959‐sss/DM4 (10 mg·kg^−1^). Strikingly, while vehicle‐treated mice were sacrificed on day 20 and 28 because their tumor volume reached the cutoff value, we observed a potent and durable antitumor response in ADC‐treated mice with initial tumor shrinkage followed by control of the disease over 48 days from the start of treatment (Fig. 3A). Despite the lower aggressiveness of the NMIBC SW780 cell model compared to T‐24 cells, we were able to obtain better tumor engraftment efficiency with the SW780 cell line. Both PBS (*n* = 6) and ADC (*n* = 6) groups were treated using the same schedule previously applied to T‐24 cells. Once again, a potent and significant antitumor response was observed (Fig. 3B) in ADC‐treated mice bearing SW780 tumors. Tumor weight at the end of the experiment (17 days from the start of treatment, defined by a tumor volume cutoff of ≈1 cm^3^ for PBS‐treated mice) was significantly lower in ADC‐treated mice (Fig. 3C, left panel). We next evaluated the ability of LGALSBP to serve as a BCa biomarker. First, we measured circulating LGALS3BP levels in mouse serum collected from PBS and ADC‐treated animals at the end of the experiment. While LGALS3BP expression levels were around 1 ng·mL^−1^ in vehicle‐treated mice, they were undetectable in ADC‐treated animals (Fig. 3C, middle panel). Moreover, a correlation trend between LGALS3BP expression levels and tumor volume in PBS‐treated xenografts was detected (Fig. 3C, right panel). Moreover, immunohistochemical analysis of SW780 tumor tissues collected at the end of the experiment confirmed a high expression of LGALS3BP both in vehicle and ADC‐treated tumors. Finally, aberrant mitotic figures were observed in ADC‐treated but not control tumor tissues, suggesting a payload‐induced G2/M cell cycle block (Fig. 3D).

### 3.4 LGALS3BP as a potential biomarker in BCa patients

We recently demonstrated that circulating LGALS3BP is a potential disease biomarker in neuroblastoma and glioblastoma [9, 14]. Given that this protein is highly expressed in bladder cancer tissues, we speculate that its circulating form could serve as a potential urinary biomarker for bladder cancer, offering a noninvasive approach for liquid biopsy. In order to increase the assay accuracy, we generated a biotinylated version of the therapeutic 1959 antibody and developed a novel in‐house ELISA for urinary LGALS3BP detection. Furthermore, to ensure high reproducibility and standardization of the assay, we purified LGALS3BP from EBNA cells stably expressing the protein, using the 1959 antibody for immunoprecipitation as described [15]. The purified protein enabled the generation of an optimal calibration curve (Fig. S1A), which was applied in all subsequent assays.

**Fig. 4:**
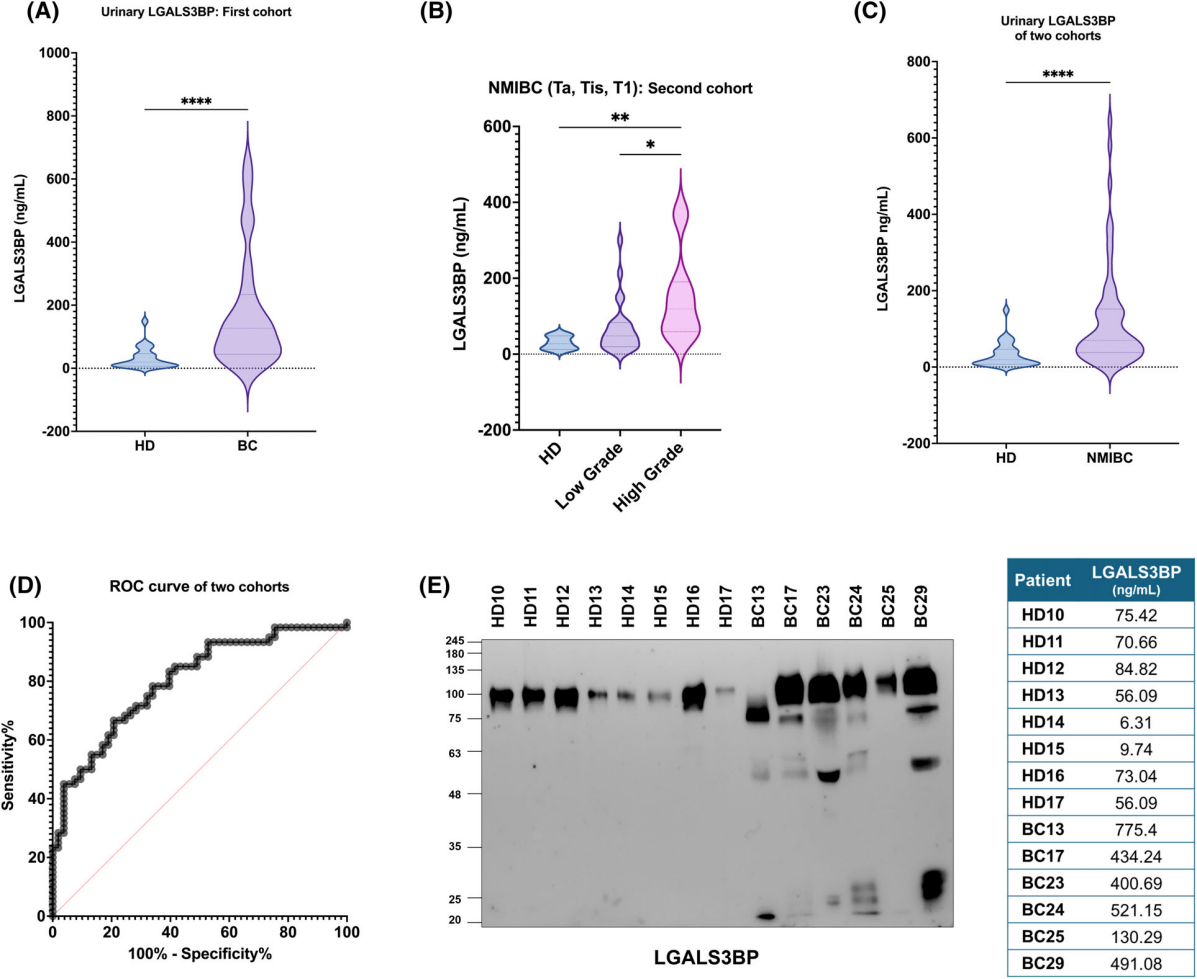
Increased Urinary LGALS3BP Expression in Bladder Cancer Patients. (A) Biotinylated 1959‐based sandwich ELISA showing LGALS3BP expression levels in urine from healthy donors (HD) (*n* = 47) or bladder cancer patients (BC) (*n* = 26) samples of a first cohort of patients. Statistical significance was obtained by using the Mann–Whitney test (****P < 0.0001). Data are presented as distribution with dotted lines corresponding to the 25th percentile, the median, and the 75th percentile from bottom to top in the violin plot. (B) Biotinylated 1959‐based sandwich ELISA showing LGALS3BP urinary expression levels (ng·mL^−1^) in HD (*n* = 6), BC low‐grade (*n* = 26), or BC high‐grade (*n* = 12) of a second cohort of patients. Statistical significance was obtained by using the Kruskal–Wallis test (**P < 0.01; *P < 0.05). Data are presented as distribution with dotted lines corresponding to the 25th percentile, the median, and the 75th percentile from bottom to top in the violin plot. (C) Biotinylated 1959‐based sandwich ELISA showing LGALS3BP urinary expression levels (ng·mL^−1^) in HD (*n* = 53) or NMIBC (*n* = 60) patients by pooling data from the two previously analyzed patient cohorts. Statistical significance was obtained by using the Mann–Whitney test (****P < 0.0001). Data are presented as distribution with dotted lines corresponding to the 25th percentile, the median, and the 75th percentile from bottom to top in the violin plot. (D) ROC curve generated by pooling data from the two previously analyzed patient cohorts. (E) Western blotting image of LGALS3BP protein pattern in urine samples from HD or BC patients. Equal amounts of samples were loaded per lane. Urinary LGALS3BP levels (ng·mL^−1^) detected through the biotinylated 1959‐based sandwich ELISA are reported in the corresponding healthy donors and BC patient samples. *n* = 2.

We first analyzed a cohort of 26 urine samples collected from bladder patients before TURBT and compared them with 47 urine samples collected from healthy donors as controls. The urinary LGALS3BP levels resulted significantly higher in bladder patients compared to healthy donors (Fig. 4A). Indeed, while the healthy donors exhibited a median LGALS3BP value of 19.29 ng·mL^−1^ (IQR 6.63–46.88 ng·mL^−1^), patient samples showed a significant increase, with a median value of 126.8 ng·mL^−1^ (IQR 44.06–234.1 ng·mL^−1^). Serum LGALS3BP levels were subsequently analyzed in a set of available control (*n* = 19) and bladder cancer patient (*n* = 57) blood samples, revealing a statistically significant elevation in patients relative to healthy individuals (Fig. S1B).

We next evaluated the potential of urinary LGALS3BP as a diagnostic biomarker and repeated the analysis in a second cohort of NMIBC patients, including low (*n* = 26) and high‐grade (*n* = 12) bladder cancer samples, and 6 samples from healthy individuals. Notably, when stratified by tumor grade, the increase in urinary LGALS3BP levels was considerably higher in high‐grade expression levels (Fig. 4B), with a median value of 119 ng·mL^−1^ (IQR 59.2–190.4 ng·mL^−1^) compared to low‐grade patients with a median value of 48.13 ng·mL^−1^ (IQR 19.87–83.56 ng·mL^−1^) and healthy donors, a median value of 27.56 ng·mL^−1^ (IQR 12.59–48.11 ng·mL^−1^). Given that all urinary samples were analyzed using the same assay and reference standard, we pooled samples from the two cohorts previously analyzed—53 healthy individuals and 60 NMIBC patients—to generate a combined ROC curve. We obtained a threshold value of 49.35 ng·mL^−1^ in healthy individuals while the average value for patients was around 120.36 ng·mL^−1^ ± 132.26 (Fig. 4C). The assay demonstrated an accuracy with 66.67% sensitivity and 79.25% specificity (Fig. 4D) with an AUC of 0.80. Furthermore, data suggested a potentially greater discriminatory power in males than in females (Fig. S1C). In order to evaluate whether urinary LGALS3BP exhibited distinct size patterns, we analyzed urine samples from healthy donors and bladder cancer (BC) patients using western blotting analysis. Interestingly, the analysis revealed a markedly different profile of LGALS3BP protein in patient urinary samples, characterized by several low molecular weight bands that were absent in healthy controls. These findings suggest that LGALS3BP may undergo aberrant glycosylation and/or proteolytic cleavage in the tumor setting (Fig. 4E).

### 3.5 LGALS3BP glycosylated status in bladder cancer

**Fig. 5:**
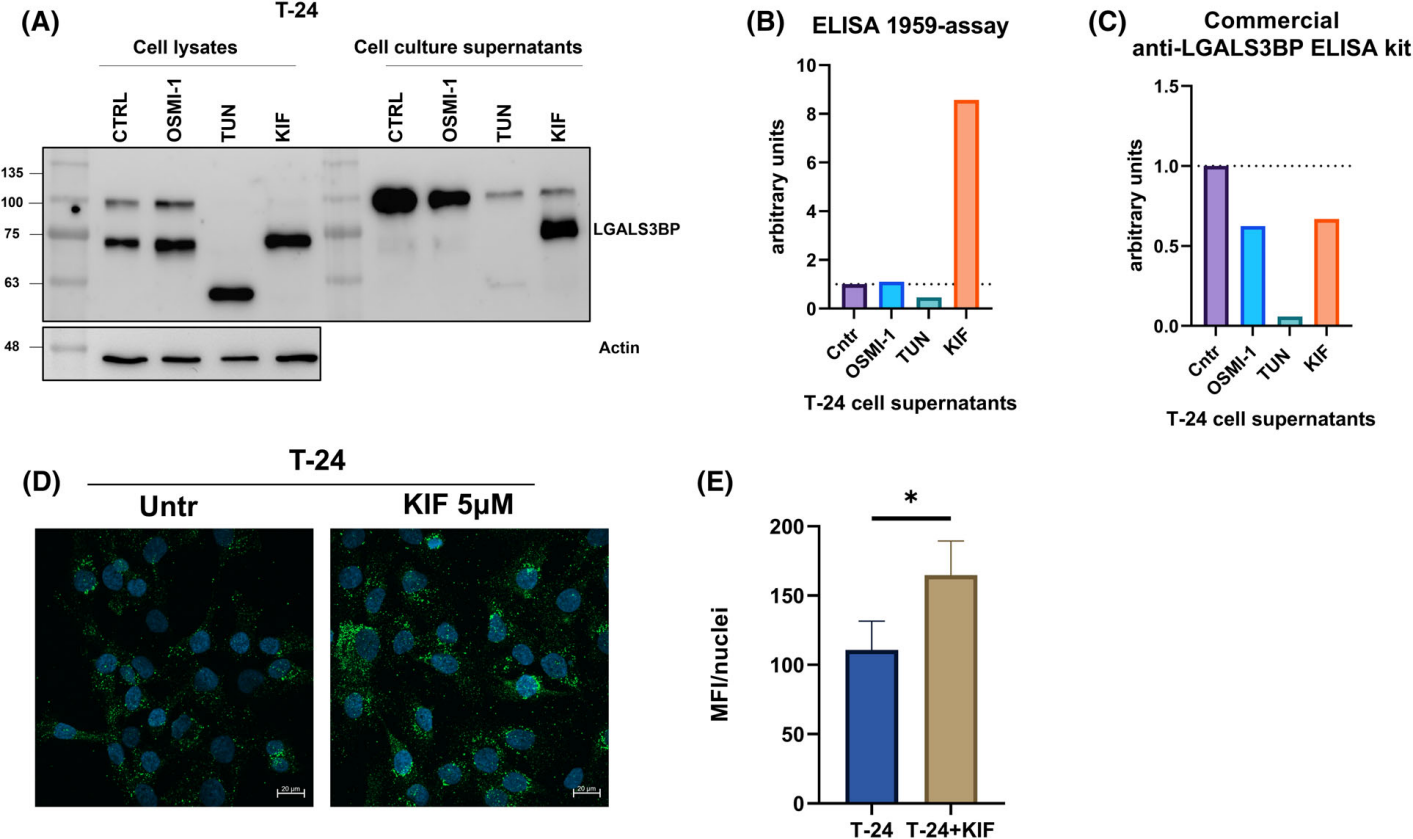
LGALS3BP glycosylation. (A) Western blot images of LGALS3BP protein pattern in cell lysates and cell culture supernatants of T‐24 cells treated or not with OSMI‐1 (50 μm), tunicamycin (TUN) (10μg·mL^−1^ ), or Kifunensine (KIF) (5 μm). Molecular weight markers are indicated on the left (kDa). *n* = 2. (B) 1959‐based sandwich ELISA (*n* = 2) and (C) commercial anti‐LGALS3BP ELISA kit (*n* = 2) for the detection of secreted LGALS3BP levels in cell culture supernatants of T‐24 cells under different treatment conditions (control, OSMI‐1, TUN, KIF). The bar graph shows normalized values expressed in arbitrary units, with all conditions normalized to the control, which is set to 1. (D) Representative confocal microscopy images of T‐24 cells under two conditions: vehicle (left) and treated with 5 μm KIF (right). Nuclei are stained in blue (DAPI), and the green signal represents LGALS3BP. Scale bar: 20 μm. Images are slices merged from z‐stack acquisition, *n* = 2. (E) Histogram representing the mean fluorescence intensity (MFI) of LGALS3BP signal per nucleus measured by confocal microscopy in T‐24 cells treated or not with KIF 5 μm (unpaired *t*‐test, *P < 0.05). *n* = 2. Data are presented as mean ± standard deviation (SD).

To better understand the glycosylation pattern of LGALS3BP in bladder cancer, we employed high‐expressing T‐24 cells as an *in vitro* model to investigate the effects of various N‐ and O‐linked glycosylation inhibitors on both the glycosylation profile and secretion of the protein. Western blot analysis confirmed that LGALS3BP undergoes glycosylation modifications in bladder cancer cells treated with glycosylation inhibitors (Fig. 5A), consistent with our previous observations in glioblastoma cells [2]. ELISA further demonstrated that protein secretion was abolished only in cells treated with Tunicamycin, an inhibitor that blocks the early steps of the N‐linked glycosylation pathway. Notably, we found that treatment with Kifunensine (KIF), a mannosidase I inhibitor that promotes the accumulation of high‐mannose enriched species, significantly enhanced the reactivity of the 1959 therapeutic antibody (Fig. 5B). Interestingly, this phenomenon is common to other cell lines from different tumors such as glioblastoma [9], neuroblastoma, and H&N cancer (Fig. S2A,B). In contrast, using a commercially available anti‐LGALS3BP ELISA, we observed that KIF treatment reduced the signal of the secreted protein (Fig. 5C and Fig. S2C,D), suggesting that the binding of high‐mannose LGALS3BP is antibody‐specific. In addition, the increase in 1959 therapeutic antibody binding to high‐mannose species was further confirmed by confocal imaging (Fig. 5D,E).

## 4 Discussion

Urothelial cancer is a malignant disease whose incidence and mortality are increasing alarmingly, requiring an urgent effort to improve the ability to make early diagnosis and therapy. Urine‐based liquid biopsy represents an emerging tool for early detection and follow‐up [16]. In fact, urine represents, for BCa, the best choice, since it is constantly in contact with the bladder mucosa and the bladder tumor; it contains fewer contaminants than blood, and it is easily obtained, not requiring particular compliance by the patient [17, 18]. Several urinary biomarkers have been investigated for their diagnostic and prognostic potential, including DNA mutations (e.g., FGFR3, TP53) [19], RNA‐based markers [20], protein markers (e.g., NMP22, BTA, UroVysion, Bladder EpiCheck) [21], and extracellular vesicles [22]. However, while the FDA has approved the urinary biomarkers BTA TRAK and NMP22, their clinical utility in detecting the disease at its onset remains limited due to the low sensitivity and specificity of these assays [23]. Therefore, despite these advancements, no single test has yet achieved a level of performance sufficient to fully resolve the challenges of early diagnosis and effective monitoring.

In the present study, we identified LGALS3BP as a potential urinary biomarker and a therapeutic target for ADC therapy. LGALS3BP is a highly glycosylated secreted protein whose levels significantly increase in cancer cells. As a circulating protein, it can be detected in body fluids, including blood, urine, and saliva, making it a promising candidate for noninvasive biomarker development. Since 1994, when two studies first identified LGALS3BP as a circulating protein overexpressed in tumors, specifically breast carcinoma and melanoma [24, 25], the interest in this molecule has steadily increased due to its documented role in regulating tumor–stroma crosstalk. The importance of LGALS3BP as a key player in tumor progression and its potential as a circulating biomarker is underscored by a growing body of research, with numerous studies published over the past decade [5, 6, 9, 11, 12, 13, 26, 27, 28, 29, 30, 31, 32]. Here, we demonstrate that LGALS3BP is overexpressed in bladder cancer, whose higher levels correlate with more advanced disease. Moreover, urinary LGALS3BP levels are significantly elevated in patients compared to healthy donors. Notably, LGALS3BP higher urinary levels are associated with high‐grade tumors in NMIBC patients, underscoring the protein's potential as a urinary biomarker. An interesting finding is that urinary levels of LGALS3BP appear to be more predictive in males than in females. The relationship between sex and LGALS3BP levels, as well as the underlying biological mechanisms, remains largely unexplored. A recent study found no significant differences in LGALS3BP serum levels based on age or sex in a healthy population [33]. In contrast, an earlier study by Melin et al. [34] in patients with type I diabetes reported that elevated LGALS3BP levels were associated with female sex. These observations support the hypothesis that protein levels in biological fluids may vary by sex, particularly in specific physiological contexts or pathological conditions.

In addition, using cellular models of bladder cancer, we focused on the LGALS3BP glycosylation pattern in cancer cells. We confirmed that the intracellular form of the protein has a size of approximately 70 kDa. However, after undergoing glycosylation, it matures into a form of around 100 kDa. In this fully processed state, the protein is secreted from the cell, as demonstrated by western blot analysis, where only the 100 kDa band is detected in the supernatant. However, it becomes clear from band sizing that the glycosylation state is cell specific, as different sizes of the mature form can be appreciated from western blotting (Fig. 2A,B). Moreover, by using different O‐ and N‐glycosylation inhibitors, we determined that only Tunicamycin, which inhibits the initial step of N‐linked glycan biosynthesis, was capable of completely blocking protein secretion. In contrast, treatment with the O‐glycosylation inhibitor (OSMI‐1) and the mannosidase I inhibitor (KIF) still resulted in the presence of LGALS3BP in the supernatant. However, in KIF‐treated cells, the protein exhibited a reduced size of approximately 70–80 kDa. This is consistent with the known effect of KIF, which prevents the trimming of mannose residues from precursor glycoproteins, leading to the accumulation of high‐mannose N‐glycans.

While we observed a slight decrease in the secreted form of LGALS3BP in the supernatants of cells treated with both OSMI‐1 and KIF—confirmed both by western blotting and a commercial ELISA—our results changed strikingly when we repeated the analysis using the ELISA based on the therapeutic antibody 1959. In this case, we observed a dramatic increase in reactivity in the samples treated with KIF, suggesting that the high‐mannose form induced by KIF treatment enhances recognition by the 1959 antibody. These results underscore that the distinct glycosylation status of LGALS3BP in tumor cells can generate specific forms that exhibit enhanced reactivity to the 1959 antibody, potentially functioning as a cancer‐specific glycoform. To further investigate this, we analyzed urine samples from both patients and healthy controls using western blotting and confirmed the presence of a distinct banding pattern in patients, likely reflecting altered glycosylation. Notably, in the patient with the highest ELISA values (BC13), we detected a band of similar size to that observed in KIF‐treated cells, further supporting the idea that high‐mannose glycoforms may be associated with tumor‐derived LGALS3BP. A recent study by Kong et al. [35] identified novel protein biomarkers in urine and blood samples from a cohort of 92 bladder cancer (BC) patients. Notably, LGALS3BP was not among the detected proteins as it was not included in the proximity extension analysis (PEA).

This circulating protein may represent a highly valuable and promising tool for improving the management of bladder cancer. It could serve both as a biomarker for the early detection of the disease monitoring as well as an indicator of therapeutic response. Designing a future study to longitudinally monitor urinary LGALS3BP levels in patients undergoing treatment or follow‐up would certainly be worthwhile. Although the present study gives promising results, it has certain limitations as well. The most significant is the small sample size. Additionally, the control population may pose a potential limitation due to imperfect age matching. Finally, the presence of high urinary protein levels in the control population made the cutoff value not sufficiently sensitive and specific for clinical translation.

In addition, further efforts will be crucial in identifying specific cancer‐associated glycosylated forms of LGALS3BP, which could significantly enhance the specificity of urinary assays. Finally, LGALS3BP or its glycosylated variants could be integrated into a multi‐panel biomarker system, improving diagnostic accuracy and follow‐up efficacy by increasing both sensitivity and specificity.

Regarding advances in precision medicine for bladder cancer, a significant milestone has been achieved with the development of an antibody‐drug conjugate (ADC) targeting Nectin‐4 of bladder cancer [36]. Indeed, Enfortumab vedotin (EV) has demonstrated potent antitumor activity in preclinical models and became the first ADC of its kind approved for clinical use for bladder cancer therapy. It has shown particularly promising results, especially in combination with immunotherapy [37]. This suggests that ADC therapy, when combined with immune checkpoint inhibitors, may represent a highly effective strategy, particularly in advanced disease settings. In this regard, 1959‐sss/DM4, the ADC developed by our group, represents a promising candidate for LGALS3BP targeted therapy in bladder cancer. In fact, this compound that has shown potent and long‐lasting antitumor activity in several preclinical models of different tumors is also particularly effective in bladder cancer as a single agent (Fig. 3A,B). We have recently identified a potential synergistic effect of this ADC in combination with the anti‐PD‐1 immune checkpoint inhibitor (ICI) in neuroblastoma [14]. This synergy appears to be driven by immunogenic cell death (ICD) induced by the DM4 payload, suggesting that 1959‐sss/DM4, in combination with ICI‐based therapies, could represent a promising therapeutic strategy for bladder cancer. Regarding the potential toxicity of DM4, it is important to note that the safety and toxicity profiles of this class of payloads have been extensively characterized in humans. For instance, the DM4‐based antibody–drug conjugate (ADC) mirvetuximab soravtansine‐gynx (MIRV), which targets folate receptor alpha, received FDA approval in late 2022 [38]. Notably, a recent report on a cohort of patients with platinum‐resistant ovarian cancer demonstrated significant antitumor activity of MIRV, along with a tolerable safety profile [39].

## 5 Conclusion

In conclusion, this study provides the first compelling evidence that the circulating protein LGALS3BP is a potential urinary biomarker for bladder cancer and, simultaneously, a promising therapeutic target for ADC‐based therapy. The findings strongly support further investigations to validate the clinical utility of LGALS3BP in bladder cancer management.

## Conflict of interest

G.S. and S.I. are shareholders of Mediapharma s.r.l. and the other authors declare no conflict of interest in this study.

## Author contributions

GS, MM, and MD designed the methodology and supervised the overall statistical analysis for the project. AP, GL, IC, EC, and AM performed key experiments of the manuscript. AP, EC, and SI contributed to writing the original draft. BF, RC, LS, JN, PCB, and MM were responsible for patient collection. RL performed immunohistochemical analysis; VDL, MD, LS, and SI provided significant insights into the interpretation of results, contributed to the study's design, secured funding, and critically revised the manuscript for important intellectual content. GS designed the study, supervised the project, and wrote the paper.

## Supporting information


**Fig. S1.** LGALS3BP as a biomarker in BCa. (A) Standard calibration curve for recombinant LGALS3BP. Optical density (OD) values were plotted against LGALS3BP concentrations (ng·mL^−1^). This standard curve was used in all subsequent quantifications for assay consistency. (B) Serum LGALS3BP levels analyzed in a set of available control (*n* = 19) and patient (*n* = 57) samples. Statistical significance was obtained by using Mann–Whitney test (****P < 0.0001). Data are presented as distribution with dotted lines corresponding to the 25th percentile, the median, and the 75th percentile from bottom to top in the violin plot. (C) Quantification of LGALS3BP levels (ng·mL^−1^) in urine samples from healthy donors (HD) (*n* = 20) and bladder cancer (BC) (*n* = 46) male patients, and from HD (*n* = 33) and BC (*n* = 14) female patients. Statistical significance was obtained by using Mann–Whitney test (*P < 0.05, ****P < 0.0001). Data are presented as distribution with dotted lines corresponding to the 25th percentile, the median, and the 75th percentile from bottom to top in the violin plot.
**Fig. S2.** LGALS3BP protein glycosylation profile in H&N and neuroblastoma cell models. Western blot images of LGALS3BP protein pattern in cell lysates and cell culture supernatants of HOC621 (*n* = 2) (A) and SKNAS (*n* = 2) (B) cells treated or not with OSMI‐1 (50 μm), tunicamycin (TUN) (10 μg·mL^−1^) or Kifunensine (KIF) (5 μm). Molecular weight markers are indicated on the left (kDa). Equal amounts of samples were loaded per lane. Actin was used as loading control. Western blot images refer to the same blot, acquired at the same time exposure. Note that one lane which served as an internal control for PNGase F activity (treatment with PNGase) has been spliced out as it is not related to this set of experiments. (C) 1959‐based sandwich ELISA (*n* = 2) and (D) commercial anti‐LGALS3BP ELISA kit (*n* = 2) for the detection of secreted LGALS3BP levels in cell culture supernatants of SKNAS and HOC621 cells under different treatment conditions (control, OSMI‐1, TUN, KIF). The bar graph shows normalized values expressed in arbitrary units, with all conditions normalized to the control, which is set to 1.
**Table S1.** Characterization of bladder cancer subtypes of a panel of human bladder cancer cell lines considered.
**Table S2.** Clinicopathological characteristics of bladder cancer patients evaluated by IHC (*n* = 29).
**Table S3.** Clinicopathological data of bladder cancer patients evaluated by ELISA.
**Table S4.** Clinicopathological data of healthy donors evaluated by ELISA.

## Data Availability

All data generated or analyzed during this study are included in this published article.
