# Supplementary material for: Glycosylated LGALS3BP is highly secreted by bladder cancer cells and represents a novel urinary disease biomarker
Source: Mol Oncol. 2025 Oct 5;20(3):823–37. doi: 10.1002/1878-0261.70140 (PMC13042393; doi:10.1002/1878-0261.70140)
Supplement: Supplementary file 1 — Fig. S1. LGALS3BP as a biomarker in BCa. (A) Standard calibration curve for recombinant LGALS3BP. Optical density (OD) values were plotted against LGALS3BP concentrations (ng·mL−1). This standard curve was used in all subsequent quantifications for assay consistency. (B) Serum LGALS3BP levels analyzed in a set of available control (n = 19) and patient (n = 57) samples. Statistical significance was obtained by using Mann–Whitney test (****P < 0.0001). Data are presented as distribution with dotted lines corresponding to the 25th percentile, the median, and the 75th percentile from bottom to top in the violin plot. (C) Quantification of LGALS3BP levels (ng·mL−1) in urine samples from healthy donors (HD) (n = 20) and bladder cancer (BC) (n = 46) male patients, and from HD (n = 33) and BC (n = 14) female patients. Statistical significance was obtained by using Mann–Whitney test (*P < 0.05, ****P < 0.0001). Data are presented as distribution with dotted lines corresponding to the 25th percentile, the median, and the 75th percentile from bottom to top in the violin plot. Fig. S2. LGALS3BP protein glycosylation profile in H&N and neuroblastoma cell models. Western blot images of LGALS3BP protein pattern in cell lysates and cell culture supernatants of HOC621 (n = 2) (A) and SKNAS (n = 2) (B) cells treated or not with OSMI‐1 (50 μm), tunicamycin (TUN) (10 μg·mL−1) or Kifunensine (KIF) (5 μm). Molecular weight markers are indicated on the left (kDa). Equal amounts of samples were loaded per lane. Actin was used as loading control. Western blot images refer to the same blot, acquired at the same time exposure. Note that one lane which served as an internal control for PNGase F activity (treatment with PNGase) has been spliced out as it is not related to this set of experiments. (C) 1959‐based sandwich ELISA (n = 2) and (D) commercial anti‐LGALS3BP ELISA kit (n = 2) for the detection of secreted LGALS3BP levels in cell culture supernatants of SKNAS and HOC621 cells under diffe [file MOL2-20-823-s001.docx]

**Supplementary Materials**

**
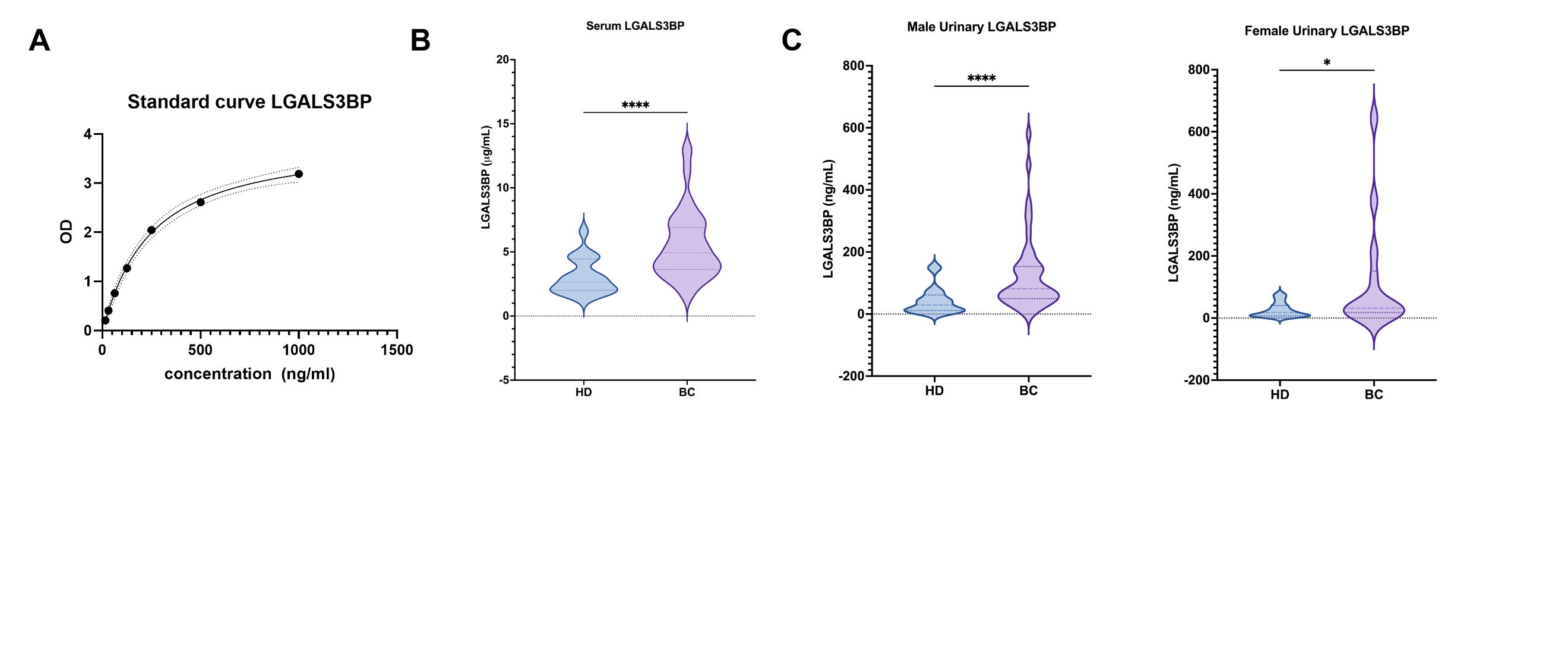
**

**Supplementary Figure S1. LGALS3BP as a biomarker in BCa. (A)** Standard calibration curve for recombinant LGALS3BP. Optical density (OD) values were plotted against LGALS3BP concentrations (ng/ml). This standard curve was used in all subsequent quantifications for assay consistency. **(B)** Serum LGALS3BP levels analyzed in a set of available control (*n*=19) and patient (*n*=57) samples. Statistical significance was obtained by using Mann-Whitney test (*****p*<0.0001). Data are presented as distribution with dotted lines corresponding to the 25^th^ percentile, the median, and the 75^th^ percentile from bottom to top in the violin plot. **(C)** Quantification of LGALS3BP levels (ng/ml) in urine samples from healthy donors (HD) (*n*=20) and bladder cancer (BC) (*n*=46) male patients, and from HD (*n*=33) and BC (*n*=14) female patients. Statistical significance was obtained by using Mann-Whitney test (**p*<0.05, *****p*<0.0001). Data are presented as distribution with dotted lines corresponding to the 25^th^ percentile, the median, and the 75^th^ percentile from bottom to top in the violin plot.

**
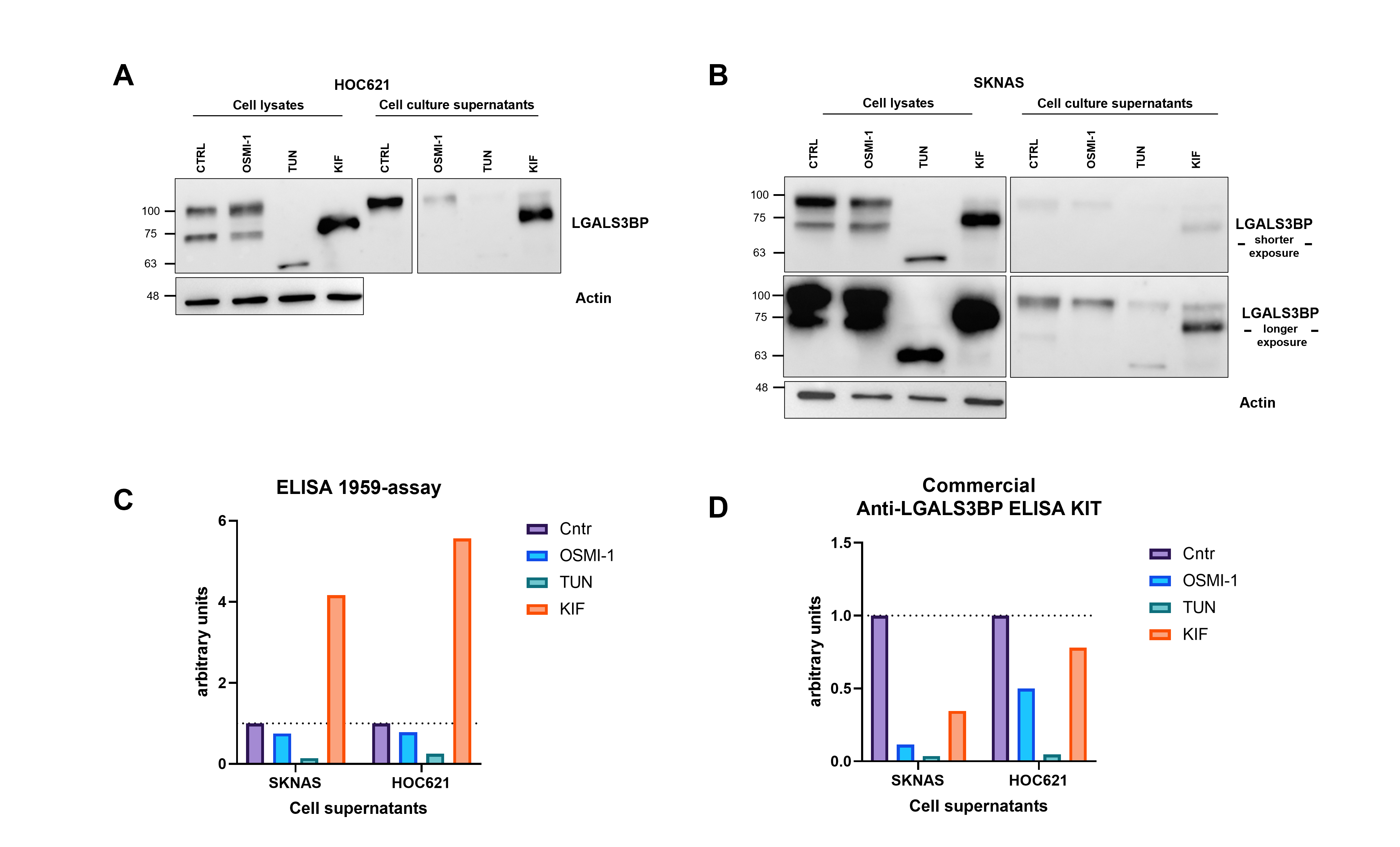
**

**Supplementary Figure S2. LGALS3BP protein glycosylation profile in H&N and neuroblastoma cell models.** Western blot images of LGALS3BP protein pattern in cell lysates and cell culture supernatants of HOC621 (*n*=2) **(A)** and SKNAS (*n*=2) **(B)** cells treated or not with OSMI-1 (50μM), tunicamycin (TUN) (10 μg/mL) or Kifunensine (KIF) (5 μM). Molecular weight markers are indicated on the left (kDa). Equal amounts of samples were loaded per lane. Actin was used as loading control. Western blot images refer to the same blot, acquired at the same time exposure. Note that one lane which served as an internal control for PNGase F activity (treatment with PNGase), has been spliced out as it is not related to this set of experiments. **(C)** 1959-based sandwich ELISA (*n*=2) and **(D)** commercial anti-LGALS3BP ELISA kit (*n*=2) for the detection of secreted LGALS3BP levels in cell culture supernatants of SKNAS and HOC621 cells under different treatment conditions (control, OSMI-1, TUN, KIF).  The bar graph shows normalized values expressed in arbitrary units, with all conditions normalized to the control, which is set to 1.

**Supplementary Table S1.** Characterization of bladder cancer subtypes of a panel of human bladder cancer cell lines considered.

| **Cell line** | **HT-1376** | **UM-UC-3** | **HT-1197** | **SW780** | **T-24** | **5637** | **TCCSUP** | **RT4** |
| --- | --- | --- | --- | --- | --- | --- | --- | --- |
| **Type** | High grade and MIBC (grade 3) | High grade and NMIBC | MIBC (grade 4) | Low grade NMIBC transitional cell carcinoma (grade 1) | High grade and MIBC transitional bladder carcinoma cells (grade 3) | Low grade and NMIBC (grade 2) | High grade and MIBC transitional cell carcinoma (grade 4) | Low grade and NMIBC transitional cell papilloma (grade 1), highly metastatic |

**Supplementary Table S2.** Clinicopathological characteristics of bladder cancer patients evaluated by IHC (n = 29).

| **Variable** | **Value (%)** |
| --- | --- |
|  |  |
| **Age at diagnosis (yr)** |  |
| **Mean ± SD** | 72.1 ± 9 |
| **Median (range)** | 71 (49-87) |
|  |  |
| **Gender** |  |
| **Male** | 24 (82.7) |
| **Female** | 5 (17.3) |
|  |  |
| **Tumor stage** |  |
| **pTx** | 1 (3) |
| **pTa** | 3 (10) |
| **pT1** | 24 (82.7) |
| **pT2** | 1 (3) |
|  |  |
| **Grading** |  |
| **Low** | 16 (55.1) |
| **High** | 13 (44.9) |

**Supplementary Table S3.** Clinicopathological data of bladder cancer patients evaluated by ELISA.

| **“SS. Annunziata” Hospital (Chieti, Italy)** | | | **Vancouver Prostate Center** | | |
| --- | --- | --- | --- | --- | --- |
| **Characteristic** | **n** | **n (%)** | **Characteristic** | **n** | **n (%)** |
| **TOT.** | 26 | 100 | **TOT.** | 38 | 100 |
| **Sex** |  |  | **Sex** |  |  |
| **Male** | 22 | 84.6 | **Male** | 27 | 71.1 |
| **Female** | 4 | 15.4 | **Female** | 11 | 28.9 |
| **Age (yr)** |  |  | **Age (yr)** |  |  |
| **60-80** | 19 | 73.1 | **39-60** | 7 | 18.4 |
| **≥80** | 7 | 26.9 | **61-91** | 31 | 81.6 |
| **Tumor stage** |  |  | **Tumor stage** |  |  |
| **1 (Ta)** | 10 | 37.5 | **1 (Ta)** | 25 | 65.8 |
| **2 (Cis)** | 1 | 3.8 | **2 (Cis)** | 8 | 21.1 |
| **3 (T1)** | 12 | 46.2 | **3 (T1)** | 5 | 13.2 |
| **4 (T2)** | 3 | 11.5 |  |  |  |
| **Grade** |  |  | **Grade** |  |  |
| **Low Grade** | 12 | 46.2 | **Low Grade** | 26 | 68.4 |
| **High Grade** | 14 | 53.8 | **High Grade** | 12 | 31.6 |

**Supplementary Table S4.** Clinicopathological data of healthy donors evaluated by ELISA.

| **HD Italy (n=47)** | | | **HD VPC (n=6)** | | |
| --- | --- | --- | --- | --- | --- |
| **Characteristic** | **n** | **n (%)** | **Characteristic** | **n** | **n (%)** |
| **TOT.** | 47 | 100 | **TOT.** | 6 | 100 |
| **Sex** |  |  | **Sex** |  |  |
| **Male** | 18 | 38.2 | **Male** | 2 | 33.3 |
| **Female** | 29 | 61.7 | **Female** | 4 | 66.7 |
| **Age (yr)** |  |  | **Age (yr)** |  |  |
| **25-50** | 24 | 51.0 | **60-65** | 4 | 66.7 |
| **51-90** | 23 | 48.9 | **66-73** | 2 | 33.3 |
